# The Impact of Aerobic Exercise and Badminton on HDL Cholesterol Levels in Taiwanese Adults

**DOI:** 10.3390/nu12051204

**Published:** 2020-04-25

**Authors:** Yasser Nassef, Kuan-Jung Lee, Oswald Ndi Nfor, Disline Manli Tantoh, Ming-Chih Chou, Yung-Po Liaw

**Affiliations:** 1 Institute of Medicine, Chung Shan Medical University, Taichung 40201, Taiwan; yasser@nassef.org; 2 Department of Public Health and Institute of Public Health, Chung Shan Medical University, Taichung 40201, Taiwan; jasminemachi@gmail.com (K.-J.L.); nforoswald2@yahoo.com (O.N.N.); tantohdisline@yahoo.com (D.M.T.); 3 Department of Family and Community Medicine, Chung Shan Medical University Hospital, Taichung 40201, Taiwan

**Keywords:** high-density lipoprotein, aerobic exercise, badminton, Taiwan Biobank

## Abstract

Elevated levels of high-density lipoprotein cholesterol (HDL-C) have been associated with a decreased risk of coronary heart disease (CHD). An active lifestyle is necessary to improve HDL-C, including (but not limited to) physical exercise. Research on the association between badminton, an intermittent exercise, and HDL-C is limited. We investigated the impact of aerobic exercise and badminton on HDL-C levels in Taiwanese adults. The sociodemographic data of 7543 participants, comprising 3472 men and 4071 women aged between 30 and 70 years, were retrieved from the Taiwan Biobank. The participants were grouped into three exercise categories—no exercise, aerobic exercise, and badminton exercise. The HDL-C levels were compared using an analysis of variance (ANOVA). Multivariate linear regression models were used to determine the associations between HDL and exercise. Comparing the other two groups to the no-exercise group, the individuals who were engaged in aerobic and badminton exercise were significantly associated with higher HDL-C levels (β = 1.4077; *p* < 0.0001 and β = 5.6052; *p* = 0.0079, respectively). Aerobic exercise and badminton were also associated with higher HDL-C levels among carriers of the lipoprotein lipase (LPL) rs328 genotypes. Aerobic exercise and regular badminton were associated with higher levels of HDL-C, with the badminton group being more significant.

## 1. Introduction

Substantial epidemiologic evidence suggests a negative linear correlation between high-density lipoprotein cholesterol (HDL-C) levels and the incidence of coronary heart disease (CHD); an inverse relationship between HDL-C and cardiovascular disease was not well established until the Framingham study in the 1970s, which identified HDL-C as a powerful risk factor inversely associated with the incidence of CHD [1]. High-density lipoprotein (HDL) is positively associated with a decreased risk of coronary heart disease (CHD). As defined by the United States National Cholesterol Education Program Adult Treatment Panel III guidelines, an HDL-C level of 60 mg/dL or greater is protective. On the other hand, a high-risk HDL-C level is described as one that is less than 40 mg/dL.

The major apolipoproteins of HDL are apolipoprotein (apo) A-I and apo A-II, the alpha-lipoproteins. Elevated concentrations of apo A-I and apo A-II are called hyperalphalipoproteinemia (HALP), which are associated with lower risk of CHD. Conversely, hypoalphalipoproteinemia increases the risk of CHD. The levels at which HDL-C confers benefit or risk are not discrete, and the cut-off points are somewhat arbitrary, especially considering that HDL-C levels are, on average, higher in women compared to men [2,3]. Hyperalphalipoproteinemia (HALP) is caused by a variety of genetic and environmental factors. Among these, plasma cholesteryl ester transfer protein (CETP) deficiency is the most important and frequent cause of HALP in Asian populations. CETP facilitates the transfer of cholesteryl ester (CE) from HDL to apo B-containing lipoproteins and is a key protein in the reverse cholesterol transport system [4].

However, environmental factors also have a significant impact on HDL-C. Smoking and obesity are the most significant risk factors associated with a lower HDL-C [5]. Besides these factors, genetic variants also have an impact on HDL-C. Certain genes play an essential role in the synthesis and metabolism of serum lipids. One of such genes is the lipoprotein lipase (LPL) gene whose variant (Rs328) has consistently been associated with lower TG and higher HDL-C levels [6,7,8]. However, the LPL rs328-GG-CG genotype was previously linked to higher concentrations of both triglycerides and HDL-C, compared to the CC genotype [9]. High LPL concentrations in serum may be atheroprotective through decreasing TG levels and increasing HDL-C levels [10]. Pathways linking LPL genotypes to HDL-C and triglycerides appear to be related [11].

Randomized controlled clinical trials have demonstrated that interventions to raise HDL-C levels are associated with reduced CHD events. Exercise is one of the lifestyle integrations that have been recommended for improving lipid fractions such as HDL-C [12]. Several studies have shown that aerobic exercise is associated with higher levels of high-density lipoprotein cholesterol. Among those researchers are Dr. Satoru Kodama (Ochanomizu University, Tokyo, Japan) and colleagues, who showed that aerobic training resulted in a 2.53 mg/dL increase in HDL-C levels; hence, by rough estimates, it could result in a 5.1% and 7.6% reduction in cardiovascular disease risk in men and women, respectively [13,14,15,16,17]. The most important element of an exercise program is the duration per session [13,16]. Aerobic exercise has also been associated with a better prognosis of cardiovascular disease [18]. Based on a previous study, intermittent exercise programs were associated with significant improvements in lipid profiles following eight weeks of training in obese children [19].

The effects of exercise behavior on the predicted cardiovascular disease (CVD) risks were found to vary depending on different factors [20]. Badminton, an indoor intermittent exercise most popular in Asia, has been shown to improve the maximum power output of regular practitioners; hence, it should be considered as a strategy for improving the health and well-being of untrained females who are currently not meeting the physical activity guidelines [21]. Outdoor exercise has been linked to air pollution and associated health issues. The respiratory physiology of exercise suggests that athletes and other exercisers may experience magnified exposure to ambient air pollution during outdoor exercise and hence should avoid exercising along roads, as ozone (O_3_) is particularly damaging to athletes [22]. As badminton is an indoor sport, playing it might reduce the harmful health effects associated with air pollution. For instance, in sedentary United Kingdom females, badminton significantly lowered some cardiovascular health markers, including the mean arterial pressure, systolic and diastolic blood pressure, and resting heart rate [21]. The findings from another study revealed that playing badminton can reduce all-cause mortality by 47% and CVD mortality risk by 59% [23].

Both aerobic exercise and badminton have positive effects on health. Several investigations have been made regarding HDL-C and aerobic exercise [16]. The results showed that HDL-C levels were more sensitive to aerobic exercise than triglycerides and other lipid fractions. As far as research on HDL-C and exercise is concerned, hardly any has been done with regards to badminton [3,21]. Because of this, we investigated the association between badminton, aerobic exercise, and HDL-C among adult Taiwanese.

## 2. Methods

### 2.1. Data Source

The data were obtained from the Taiwan Biobank, a national health resource. The Biobank contains the genetic information of over 200,000 ethnic Taiwanese residents aged 30–70 years [24]. Presently, there are 29 recruitment centers, with each city or county having at least one. The recruitment methods in the Taiwan Biobank are in accordance with the relevant guidelines and regulations. Written informed consent is obtained from all of the participants prior to data collection. The data are collected through questionnaires as well as physical and biochemical examinations. The study was conducted in accordance with the Declaration of Helsinki, and the protocol was approved by the Institutional Review Board of Chung Shan Medical University.

### 2.2. Study Participants

Overall, 7543 individuals, consisting of 3472 men and 4071 women aged 30–70 years, were recruited. Their demographic (age, sex, body mass index (BMI), waist–hip ratio (WHR), and body fat), biochemical (HDL-C), and lifestyle (physical activity, coffee drinking, smoking, alcohol consumption, and betel nut chewing) data were retrieved from the database. The participants were categorized based on exercise status—no exercise (did not exercise at all during the last three months), aerobic exercise (did at most 3 types, which included jogging, strolling, swimming, yoga, taijiquan, biking, and aerobic dance, and badminton (that is, only regular badminton, 30 min per session, and at least 3 times per week in the last 3 months)).

### 2.3. Statistical Analysis

The data were managed and analyzed using the SAS 9.4 software (SAS Institute, Cary, NC). One-way analysis of variance (ANOVA) was used to compare the HDL-C levels in the various exercise groups. To further compare differences among groups, a post-hoc analysis was performed using the Scheffe test. Multivariate linear regression models were used to determine the association between HDL-C and exercise. The data were presented as mean ± standard error (SE) for the continuous variables.

## 3. Results

Table 1 shows the baseline characteristics of the participants in different exercise groups. The participants comprised 5053 participants who did not exercise, 2461 who did aerobic exercise, and 29 who played badminton. The mean ± SE HDL-C in various exercise groups for no exercise, aerobic exercise, and badminton were 47.36 ± 0.22 (men) and 57.78 ± 0.25 (women), 49.97 ± 0.35 (men) and 59.24 ± 0.38 (women), and 51.05 ± 2.08 (men) and 72.50 ± 7.63 (women), respectively. Among both the men and women, there were significant differences in HDL-C between the exercise groups. Table 2 shows two separate models demonstrating the association of HDL-C with aerobic exercise (model 1) and badminton exercise (model 2). After adjusting for confounders, HDL-C was positively associated with aerobic exercise (β = 1.3997, *p* < 0.0001) and badminton (β = 5.6585, *p* = 0.0061) when compared to no exercise. Table 3 shows the association between HDL-C and exercise, with both aerobic exercise and badminton included in the same model. There were positive associations of HDL-C with aerobic exercise and badminton (β = 1.4077, *p* < 0.0001 and β = 5.6052, *p* = 0.0079, respectively). The Rs328 CG/GG genotype was associated with increased levels of HDL-C (β = 2.3521, *p* < 0.0001) when aerobic exercise and badminton were both included in the model. HDL-C was negatively associated with male sex (β = −9.6558, *p* < 0.0001), WHR (β = −3.0905, *p* < 0.0001), body fat (β = −2.0067, *p* < 0.0001), overweight (β = −4.1867, *p* < 0.0001), obesity (β = −6.7435, *p* < 0.0001), current smoking (β = −3.4080, *p* < 0.0001), and current vegetarian diet (β = −6.0259, *p* < 0.0001). However, it was positively associated with coffee drinking, age, being underweight, and current drinking. 

Compared with no exercise, aerobic exercise and badminton were associated with higher HDL-C levels among carriers of rs328 genotypes (Table 4). The β-values were higher for badminton than for aerobic exercise (i.e., 4.6375 vs. 1.2489 for the CC genotype and 8.6775 vs. 2.1472 for the CG + GG genotype), and the tests for trend were statistically significant (*p* < 0.05). 

## 4. Discussion

To our knowledge, this is the first Asian study that has investigated the effect of badminton on HDL-C. There were significant associations between HDL-C and aerobic exercise and badminton. Several studies have been carried out on the association between HDL-C and exercise only. However, the results have not been consistent. While some demonstrated that regular exercise could significantly raise the serum levels of HDL-C [15,16,25], others showed no significant changes [26,27,28,29]. In our study, the badminton effect on HDL-C was stronger than the aerobic exercise. The mechanisms underlying these associations are still unclear. However, these effects might be linked to a higher expression of liver ATP-binding cassette transporters A-1 (ABCA1) [30], caused by the upregulation of the liver X receptor (LXR) [18]. This may improve the reverse cholesterol transport (RCT) process and can cause more cholesterol to be transported to the liver via HDL.

Several studies have reported that different exercise types can influence cholesterol and may change personal health status [16,17,25,28,31]. As far as the association of badminton with HDL-C is concerned, there is limited research in this area. A study conducted in the United Kingdom showed significant associations between badminton and cardiovascular health markers [21]. Nonetheless, how different exercise types influence the risk of cardiovascular diseases is yet to be fully understood. Similar to our results, Sasaki and colleagues found that long-term aerobic exercise was associated with an increase in HDL-C and weight reduction in obese children [32]. Research focused on Taiwanese adults also discovered that regular weekly exercise durations of <2.5 and ≥2.5 h were both positively associated with HDL-C in both sexes. However, the associations were stronger in males than females [13].

It is worth mentioning that significant associations were found between exercise and HDL-C based on age, gender, obesity, blood pressure, blood cholesterol, smoking, and alcohol drinking, some of which have served as risk factors for cardiovascular diseases [33,34,35,36]. Compared to several previous studies, this study had a larger sample size. To better understand the relationship between exercise and HDL-C, participants were grouped into different exercise categories—no exercise (did not do exercise at all during the last three months), aerobic exercise (three different types of any exercise at least, regularly performed three times a week for at least 30 min each session, where badminton was not one of these three exercises), and badminton (only practicing badminton regularly in the last three months, and not any other type of sports). So far, such stratifications had not been made in studies conducted in Asia, particularly in Taiwan. The addition of LPL Rs328 in the model did not modulate the association between HDL-C and exercise modality. However, we found a significant association between its genotypes and HDL-C levels. For instance, rs328CG/GG polymorphism influenced the effect of current alcohol drinking and smoking on HDL-C levels. That is, among the rs328CG/GG carriers, current alcohol drinking was associated with increased HDL-C levels (β = 4.8369, *p* < 0.0001), while current smoking was linked to lower HDL-C (β = −3.4325, *p* = 0.0009). The possible mechanisms underlying these associations remain to be clarified. The effects of age and coffee drinking on HDL-C of rs328CG/GG carriers were not significant. The study is limited in that the sample size for the badminton players was small. This is because these players included those that were restricted only to badminton and nothing else in the last three months.

In conclusion, aerobic exercise and regular badminton were associated with higher levels of HDL-C, with the badminton group being more significant. Further investigations with large-scale sample sizes are recommended in order to make a stronger and definite conclusion regarding the link between HDL-C and badminton in particular.

## Figures and Tables

**Table 1:** Basic data and mean high-density lipoprotein cholesterol (HDL-C) (mg/dL) by exercise type.

Variable	No Exercise		Aerobic Exercise		Badminton		*p*-Value
	(*n* = 5053)		(*n* = 2461)		(*n* = 29)		
	*n* (%)	Mean ± SE	*n* (%)	Mean ± SE	*n* (%)	Mean ± SE	
**rs328**							
CC	4069(80.53)	52.64 ± 0.20 ^a^	1997(81.15)	54.42 ± 0.30 ^b^	22(75.86)	54.73 ± 3.76 ^ab^	<0.0001
CG+GG	984(19.47)	54.68 ± 0.42 ^a^	464(18.85)	57.00 ± 0.64 ^b^	7(24.14)	64.00 ± 4.22 ^ab^	0.0020
CG	935(18.50)	54.56 ± 0.43 ^a^	438(17.80)	56.97 ± 0.65 ^b^	7(24.14)	64.00 ± 4.22 ^ab^	0.0017
GG	49(0.97)	56.96 ± 1.85	26(1.06)	57.58 ± 3.30	-	-	0.8603
**Sex**							
Female	2753(54.48)	57.78 ± 0.25 ^a^	1310(53.23)	59.24 ± 0.38 ^b^	8(27.59)	72.50 ± 7.63 ^c^	<0.0001
Male	2300(45.52)	47.36 ± 0.22 ^a^	1151(46.77)	49.97 ± 0.35 ^b^	21(72.41)	51.05 ± 2.08 ^ab^	<0.0001
**Waist–hip ratio**							
Male < 0.9; female < 0.8	1986(39.30)	54.64 ± 0.30 ^a^	929(37.75)	56.31 ± 0.46 ^b^	15(51.72)	57.07 ± 3.85 ^ab^	0.0076
Male ≥ 0.9; female ≥ 0.8	3067(60.70)	52.00 ± 0.23 ^a^	1532(62.25)	54.05 ± 0.34 ^b^	14(48.28)	56.86 ± 5.05 ^ab^	<0.0001
**Body fat (%)**							
Male < 25; female < 30	2580(51.06)	55.37 ± 0.27	1375(55.87)	56.30 ± 0.38	16(55.17)	61.13 ± 4.69	0.0390
Male ≥ 25; female ≥ 30	2473(48.94)	50.59 ± 0.24 ^a^	1086(44.13)	53.14 ± 0.38 ^b^	13(44.83)	51.85 ± 3.44 ^ab^	<0.0001
**Coffee drinking**							
Yes	3350(66.30)	54.39 ± 0.33 ^a^	1666(67.70)	55.70 ± 0.49 ^b^	20(68.97)	56.67 ± 6.57 ^ab^	<0.0001
No	1703(33.70)	52.35 ± 0.22	795(32.30)	54.53 ± 0.33	9(31.03)	57.10 ± 3.49	0.0740
**Age**							
30–40	1818(35.98)	53.60 ± 0.30	332(13.49)	55.46 ± 0.73	10(34.48)	57.60 ± 5.90	0.0403
41–50	1595(31.57)	52.88 ± 0.33 ^a^	583(23.69)	54.90 ± 0.55 ^b^	9(31.03)	61.00 ± 6.54 ^ab^	0.0016
51–60	1138(22.52)	52.87 ± 0.39 ^a^	925(37.59)	54.69 ± 0.45 ^b^	6(20.69)	48.00 ± 3.84 ^ab^	0.0050
61–70	502(9.93)	51.83 ± 0.57 ^a^	621(25.23)	54.93 ± 0.56 ^b^	4(13.79)	59.75 ± 5.99 ^ab^	0.0004
**BMI**							
Normal	2364(46.78)	57.40 ± 0.27 ^a^	1220(49.57)	58.79 ± 0.39 ^b^	14(48.28)	60.64 ± 5.29 ^ab^	0.0087
Underweight	159(3.15)	64.99 ± 1.14	43(1.75)	67.98 ± 2.39	-	-	0.2355
Overweight	1460(28.89)	49.76 ± 0.30 ^a^	778(31.61)	52.17 ± 0.44 ^b^	9(31.03)	56.67 ± 3.94 ^ab^	<0.0001
Obese	1070(21.18)	46.07 ± 0.31	420(17.07)	47.36 ± 0.50	6(20.69)	48.83 ± 5.61	0.0775
**Smoking**							
Never	3861(76.41)	54.74 ± 0.21 ^a^	1947(79.11)	56.39 ± 0.31 ^b^	22(75.86)	59.68 ± 3.78 ^ab^	<0.0001
Quit	505(9.99)	48.58 ± 0.51	329(13.37)	49.95 ± 0.64	5(17.24)	51.80 ± 3.68	0.2129
Current	687(13.60)	46.72 ± 0.44	185(7.52)	48.05 ± 0.92	2(6.90)	40.00 ± 2.00	0.2693
**Drinking**							
Never	4563(90.30)	53.34 ± 0.19 ^a^	2209(89.76)	55.37 ± 0.29 ^b^	25(86.21)	58.80 ± 3.43 ^ab^	<0.0001
Quit	114(2.26)	44.67 ± 0.96	94(3.82)	47.34 ± 1.13	-	-	0.0710
Current	376(7.44)	51.86 ± 0.70	158(6.42)	52.84 ± 1.01	4(13.79)	45.50 ± 2.50	0.4497
**Vegetarian**							
No	4543(89.91)	53.13 ± 0.19 ^a^	2251(91.47)	55.22 ± 0.29 ^b^	26(89.66)	57.15 ± 3.45 ^ab^	<0.0001
Former	258(5.11)	54.37 ± 0.92	104(4.23)	53.44 ± 1.33	2(6.90)	57.00 ± 1.00	0.8250
Current	252(4.99)	49.89 ± 0.71	106(4.31)	49.69 ± 1.08	1(3.45)	52.00	0.9698

Variables are expressed as numbers (%) and mean ± SE and compared using the Scheffe test (post-hoc test). Means with a different superscript (a, b) are significantly different between the exercise groups. SE: standard error; BMI: body mass index (measured in kg/m^2^); *n*: number.

**Table 2:** Association of HDL with aerobic exercise (model 1) and badminton (model 2).

Variables	Model 1			Model 2		
	β	SE	*p*-Value	β	SE	*p*-Value
Exercise (Ref: no exercise)						
Aerobic exercise	1.3997	0.2945	<0.0001	-	-	-
Badminton	-	-	-	5.6585	2.0616	0.0061
rs328 (Ref: CC)						
CG + GG	2.3351	0.3310	<0.0001	2.1152	0.3920	<0.0001
Sex (Ref: Female)						
Male	−9.6065	0.3437	<0.0001	−9.7497	0.4074	<0.0001
Waist–hip ratio (Ref: Male < 0.9; Female < 0.8)						
Male ≥ 0.9; Female ≥ 0.8	−3.0988	0.3096	<0.0001	−2.8774	0.3675	<0.0001
Body fat rate (Ref: Male < 25; Female < 30)						
Male ≥25; Female ≥ 30	−1.9633	0.3428	<0.0001	−2.1231	0.4099	<0.0001
Coffee (Ref: No)						
Yes	1.2102	0.2800	<0.0001	1.1652	0.3325	0.0005
Age (Ref: (30–40))						
41–50	0.6271	0.3502	0.0734	0.8015	0.3846	0.0372
51–60	1.1843	0.3685	0.0013	1.2700	0.4284	0.0030
61–70	1.2547	0.4466	0.0050	0.7730	0.5716	0.1763
BMI (Ref: Normal)						
Underweight	5.7177	0.8290	<0.0001	5.3988	0.9199	<0.0001
Overweight	−4.2243	0.3476	<0.0001	−4.1937	0.4219	<0.0001
Obese	−6.7836	0.4444	<0.0001	−6.5573	0.5266	<0.0001
Smoking (Ref: Never)						
Former	−0.4754	0.4606	0.3021	−0.3537	0.5650	0.5313
Current	−3.4372	0.4607	<0.0001	−3.3528	0.5198	<0.0001
Drinking (Ref: Never)						
Former	−0.9769	0.8229	0.2352	−0.6859	1.0817	0.5261
Current	4.5933	0.5418	<0.0001	4.7457	0.6295	<0.0001
Vegetarian (Ref: No)						
Former	−0.0541	0.6115	0.9295	0.5785	0.7065	0.4129
Yes	−6.0180	0.6175	<0.0001	−5.3671	0.7201	<0.0001

SE = standard error; β = beta value; ref. = reference; model 1 = association between HDL-C and aerobic exercise; model 2 = association between HDL-C and badminton.

**Table 3:** Association between HDL-C and exercise type (aerobic exercise and badminton are included in the same model).

	β	SE	*p*-Value
Exercise (Ref: no exercise)			
Aerobic exercise	1.4077	0.2948	<0.0001
Badminton	5.6052	2.1100	0.0079
rs328 (Ref: CC)			
CG+GG	2.3521	0.3306	<0.0001
Sex (Ref: Female)			
Male	−9.6558	0.3435	<0.0001
WHR (Ref: Male<0.9; Female<0.8)			
Male ≥ 0.9; Female ≥ 0.8	−3.0905	0.3095	<0.0001
Body fat rate (Ref: Male < 25; female < 30)			
Male ≥ 25; female ≥ 30	−2.0067	0.3428	<0.0001
Coffee (Ref: No)			
Yes	1.1931	0.2797	<0.0001
Age (Ref:30–40)			
41–50	0.6273	0.3498	0.0730
51–60	1.1515	0.3682	0.0018
61–70	1.2305	0.4461	0.0058
BMI (Ref: Normal)			
Underweight	5.7009	0.8298	<0.0001
Overweight	−4.1867	0.3473	<0.0001
Obese	−6.7435	0.4442	<0.0001
Smoking (Ref: Never)			
Former	−0.4787	0.4596	0.2977
Current	−3.4080	0.4603	<0.0001
Drinking (Ref: Never)			
Former	−0.9741	0.8236	0.2369
Current	4.4928	0.5402	<0.0001
Vegetarian (Ref: No)			
Former	−0.0589	0.6104	0.9231
Current	−6.0259	0.6174	<0.0001

**Table 4:** Association between HDL-C and exercise type (aerobic exercise and badminton are included in the same model) stratified by rs328 genotypes.

	CC			CG + GG		
	β	SE	*p*-Value	β	SE	*p*-Value
Exercise (Ref: no exercise)						
Aerobic exercise	1.2489	0.3279	0.0001	2.1472	0.6766	0.0015
Badminton	4.6375	2.4277	0.0561	8.6775	4.2688	0.0423
*p* for trend	<0.0001			0.0004		

Adjusted for age, sex, waist–hip ratio (WHR), body fat rate, coffee drinking, body mass index (BMI), smoking, drinking, and vegetarian diet. β: beta coefficient; SE: standard error.
